# Nanopore-based metagenomic sequencing for the rapid and precise detection of pathogens among immunocompromised cancer patients with suspected infections

**DOI:** 10.3389/fcimb.2022.943859

**Published:** 2022-09-20

**Authors:** Qingmei Deng, Yongqing Cao, Xiaofeng Wan, Bin Wang, Aimin Sun, Huanzhong Wang, Yunfei Wang, Hongzhi Wang, Hongcang Gu

**Affiliations:** ^1^ Anhui Province Key Laboratory of Medical Physics and Technology, Institute of Health and Medical Technology, Hefei Institutes of Physical Science, Chinese Academy of Science, Hefei, China; ^2^ Science Island Branch, Graduate School of University of Science and Technology of China, Hefei, China; ^3^ Hefei Cancer Hospital, Chinese Academy of Sciences, Hefei, China; ^4^ The Cancer Hospital of the University of Chinese Academy of Sciences, Institute of Basic Medicine and Cancer, Chinese Academy of Sciences, Hangzhou, China; ^5^ Zhejiang ShengTing Biotechnology Company, Hangzhou, China

**Keywords:** cancer, pathogen detection, infections, metagenomic sequencing, nanopore amplicon sequencing

## Abstract

Cancer patients are at high risk of infections and infection-related mortality; thereby, prompt diagnosis and precise anti-infectives treatment are critical. This study aimed to evaluate the performance of nanopore amplicon sequencing in identifying microbial agents among immunocompromised cancer patients with suspected infections. This prospective study enlisted 56 immunocompromised cancer patients with suspected infections. Their body fluid samples such as sputum and blood were collected, and potential microbial agents were detected in parallel by nanopore amplicon sequencing and the conventional culture method. Among the 56 body fluid samples, 47 (83.9%) samples were identified to have at least one pathogen by nanopore amplicon sequencing, but only 25 (44.6%) samples exhibited a positive finding by culture. Among 31 culture-negative samples, nanopore amplicon sequencing successfully detected pathogens in 22 samples (71.0%). Nanopore amplicon sequencing showed a higher sensitivity in pathogen detection than that of the conventional culture method (83.9% vs. 44.6%, P<0.001), and this advantage both existed in blood samples (38.5% vs. 0%, P=0.039) and non-blood samples (97.7% vs. 58.1%, P<0.001). Compared with the culture method, nanopore amplicon sequencing illustrated more samples with bacterial infections (P<0.001), infections from fastidious pathogens (P=0.006), and co-infections (P<0.001). The mean turnaround time for nanopore amplicon sequencing was about 17.5 hours, which was shorter than that of the conventional culture assay. This study suggested nanopore amplicon sequencing as a rapid and precise method for detecting pathogens among immunocompromised cancer patients with suspected infections. The novel and high-sensitive method will improve the outcomes of immunocompromised cancer patients by facilitating the prompt diagnosis of infections and precise anti-infectives treatment.

## Introduction

Cancer causes serious harm to human health worldwide and has long been a significant challenge in biomedical and clinical research (Siegel et al., 2021). Infections are common among cancer patients and are accountable for most noncancer causes of death (Zaorsky et al., 2017; Lehrnbecher et al., 2021). Some clinical observational studies revealed that cancer patients with common bacterial infections had a 2-fold increased risk of death than those without infections (Brand et al., 2016; Hjelholt et al., 2021). Anti-cancer therapies such as conventional chemotherapy, radiotherapy, and treatments using immune checkpoint inhibitors (ICIs) or targeted drugs can result in the immunocompromised status among cancer patients, increasing the risk of infections (Eilers et al., 2010; Weil et al., 2018; Malek et al., 2021). Because of the high prevalence and the adverse outcomes, infections represent a substantially challenging issue in the management of cancer patients, especially for those with immunocompromised status (Casto et al., 2021). Therefore, there is currently an urgent need to improve both the surveillance and treatments for infections among cancer patients.

Early diagnosis and effective therapy with precise anti-infectives are critical to improving the outcomes of patients suffering from infections (Van Cutsem et al., 2016; Garcia-Vidal et al., 2021). Conventional culture-based pathogen detection methods have defects such as lower sensitivity and longer turnaround time, thus having limited roles in rapidly and accurately detecting pathogens (Peri et al., 2022). Recently, multiple novel microbiological detection techniques have been developed, and some show great potential in the diagnosis of infections in terms of turnaround time, accuracy, and antimicrobial resistance (Henderson et al., 2021; Peri et al., 2021b). The nanopore-sequencing developed based on the Oxford Nanopore Technologies is one representative of the third-generation sequencing techniques, and its role in the identification of pathogens has gained increasing attention in recent years (Ferreira et al., 2021). Nanopore-sequencing presents many significant merits, such as higher sensitivity, better accuracy, and less turnaround time than the routine culture-based methods, thus enabling patients to receive earlier and more precise antimicrobial therapies (Dippenaar et al., 2021; Noone et al., 2021). However, the application of nanopore amplicon sequencing in detecting pathogens among immunocompromised cancer patients has not been evaluated. Cancer patients, especially those with immunocompromised status, are at high risk of infections. The early and accurate identification of pathogens by nanopore amplicon sequencing will undoubtedly benefit those patients. This study evaluated its performance in detecting pathogens among immunocompromised cancer patients with suspected infections, and a side-by-side comparison with the culture method was demonstrated.

## Methods

### Patient enrolment

This prospective study was designed to evaluate the performance of nanopore amplicon sequencing in detecting pathogens among immunocompromised cancer patients with suspected infections. The schematic workflow of pathogen detection by nanopore-sequencing was shown in **Supplementary Figure 1**. Hospitalized cancer patients with suspected infections were routinely screened in those two hospitals between January 2021 and July 2021. To be enrolled in this study, patients must meet the following inclusion criteria: 1) a precise diagnosis of cancer; 2) symptoms of infections during this hospital stay; 3) sufficient amount of body fluid samples such as sputum, blood and bronchoalveolar lavage fluid (BALF) for testing by nanopore-sequencing and the conventional culture-based method; 4) a written informed consent. Patients with the following conditions were excluded: 1) no evidence of infections; 2) the sample volume not enough for testing; 3) missing one test result; 4) no written informed consent. This study was approved by the Ethics Committee of the Hefei Cancer Hospital of Chinese Academy of Science and the Cancer Hospital of the University of Chinese Academy of Sciences. This study followed guidelines established by the Helsinki Declaration (World Medical Association, 2013).

### Sample collection

Body fluid samples including BALF, sputum, abscess, peritoneal fluid, pleural fluid, urine, blood, and other fluids were collected in sterile tubes from patients. All body fluid samples must meet the criteria for clinical examination. The culture assays for bacteria and fungi were performed routinely in-house at hospitals. Samples were processed immediately unless otherwise specified.

### Conventional culture

Potential pathogens were detected routinely by the standard culture methods for detection of pathogens at the Laboratory Medicine Department. Generally, clinical specimens were cultured on blood agar media, MacConkey agar media, chocolate agar media, and Sabouraud agar media (Babio Biotechnology, China). For the cultivation of anaerobic and facultatively anaerobic bacteria, samples were incubated for 18-72 hours at 36.5 ± 0.5°C in 5%CO2. For the cultivation of anaerobic bacteria, samples were incubated in anaerobic bags for 18-72 hours at 36.5 ± 0.5°C. For fungi culture, samples were incubated for 7 days at 28 ± 0.5°C. For a blood culture, samples were incubated for 5 days at 35.5 ± 0.5°C. Pathogen identification was performed with BacT/ALERT 3D Automated Microbial Detection System (bioMérieux, Inc., France) and/or VITEK^®^ 2 COMPACT Automated Microbial Identification System (bioMérieux, Inc., USA). Owing to the high requirements in specialized facilities and biosafety for virus culture and the lower sensitivity, culture-based virus detection was not routinely carried out in these two hospitals (Leland and Ginocchio, 2007; Cassedy et al., 2021).

### DNA extraction and polymerase chain reaction amplification

DNA from samples was extracted using the QIAamp DNA Microbiome Kit (Cat. No. 51707, Qiagen, Hilden, Germany) per the manufacturer’s instructions. Viral DNA was extracted from clinical samples with conventional DNA extraction protocols. The extracted DNA was used for PCR amplification of the 16S rDNA regions and fungal internal transcribed spacer (ITS) regions using a 16S rDNA PCR kit and a fungal ITS PCR kit. To reduce the complexity of library preparation and control time cost, PCR amplification of the 16S rDNA regions and fungal ITS regions was performed in one tube with an optimized primer mixture, in which the primer ratios of bacteria and fungi were set at 5:2. For the detection of possible DNA viruses in clinical specimens, PCR amplification of key genes of 10 common DNA viruses such as Epstein-Barr virus (EBV) and human cytomegalovirus (HCMV) was performed separately. The primers details used for PCR amplification were shown in the **Supplementary Table 1**. The PCR conditions were shown as follows: initiation denaturation step at 98°C for 3min, then six cycles of 95°C for 15s/66°C for 60s/72°C for 30s, then another 29 cycles of 95°C for 15s/61°C for 60s/72°C for 30s, and a final extension step of 72°C for 5min. All PCRs were performed on an ABI 2720 Thermal Cycler (Cat. No. 435659; ABI, California, USA).

### Library preparation

Products from PCR experiments were then purified with 0.8× AMPure beads for Nanopore Barcode PCR step. The purified PCR products were used for Nanopore barcode PCR according to PCR Barcoding Expansion Pack 1-96 (EXP-PBC096). The Nanopore barcode PCR products were purified with 0.6× AMPure beads, and each purified barcode PCR product was pooled with equal amounts for nanopore library preparation. The purified PCR products were used for subsequent library preparation, and it was carried out using the DNA library preparation kit following the manufacturer’s instructions (Cat. No. SQK-LSK109, Oxford Nanopore Technologies, Oxford, UK). The library was further eluted with 15μl TE buffer for quantified. For each sample, two parallel libraries were prepared and sequenced separately including one for detecting bacteria and fungi and one for detecting DNA viruses (Karamitros and Magiorkinis, 2018).

### Nanopore sequencing

The purified libraries were loaded on a Nanopore flow cell (R9.4.1) on MinION platform after chip priming and were sequenced using GridION platform, and 80 fmol final library for each sample was loaded (Jain et al., 2015). Real-time data acquisition was performed with the MinKNOW software. Sequenced reads were then used for subsequent analyses of pathogen identification.

### Identification of pathogens

Sequenced reads were analyzed by the What’s In My Pot (WIMP) workflow *via* EPI2ME (Juul et al., 2015; Sakai et al., 2019). The reads less than 200 bp and greater than 2500 bp were filtered, reads derived from human DNA were removed by searching each read against the human genome using minimap2 (Li, 2018), and the remaining reads were aligned to the pathogens databases in National Center for Biotechnology Information (NCBI). Pathogens were classified at the species level based on the percentage of coverage and identity. Generally, those microorganisms within the top 10 pathogens ranked by aligned reads and with at least 10 aligned reads or a relative abundance score >0.5% were classified as possible pathogens and were subjected to further evaluation. For some special pathogens such as *Mycobacterium tuberculosis*, specific criteria were adopted.

### Limit of detection

To evaluate the limit of detection (LoD) of nanopore amplicon sequencing for bacteria and fungi in clinical body fluid, we spiked the two most common clinical pathogens, *Escherichia coli and Candida albicans*, into the negative sputum specimen of healthy donors, starting from 10^6^ copies/ml in a series of 10-fold dilution gradient to 10^2^ copies/ml. Pathogen detection for each concentration was performed with two replicates for accuracy and reproducibility. The positive threshold of effective reads for analysis were set to coverage ≥85% and identity ≥ 90%. The limit of detection was defined as the concentration at which the total valid reads of sequencing was ≥30,000, and the reads of the pathogens were ≥10 in both replicates.

### Validation of identified pathogens

The presence of some clinically significant pathogens in clinical samples were further validated by quantitative reverse transcription PCR (RT-qPCR) assays or Sanger sequencing. Those validation experiments were performed with the residual DNA extracted from clinical samples. In general, pathogens such as *Mycobacterium tuberculosis* and fungi such as *Candida glabrata*, *Candida albicans*, *Candida tropicalis*, *Pneumocystis jirovecii* and *Aspergillus niger* would be confirmed with genus- or species-specific commercial RT-PCR diagnostic kits in accordance with the manufacturer’s instructions. For DNA viruses, Sanger sequencing would be used to validate the presence of those DNA viruses in the clinical samples.

### Statistical analyses

Normally distributed continuous data were shown as mean with standard deviation (SD), while those data with non-normal distribution were shown as median with interquartile range (IQR). For normally distributed data, differences between groups were calculated by t-test; while for data of non-normal distribution, differences between groups were calculated by Mann-Whitney U test. Count data were displayed as a number with percentage, and differences between groups were evaluated by Fisher’s exact test or Chi-square test. The performance of those two methods in detecting pathogens was compared, and subgroups stratified by pathogen type and sample type were performed. Data were analyzed by R software (Version 3.6.1, R Foundation). A two-sided P < 0.05 was considered to be statistically significant.

## Results

### Characteristics of patients

A total of 56 immunocompromised cancer patients with suspected infections were enrolled in this study (**Table 1**). Among those patients, the most common cancer type was lung cancer (22 cases, 39.3%), followed by esophageal cancer (7 cases, 12.5%), cervical cancer (5 cases, 8.9%), and liver cancer (5 cases, 8.9%). The treatments for those patients were also diverse, including chemotherapy for 41 cases (73.2%), radiotherapy applied to 13 patients (23.2%), ICIs therapy among 18 cases (32.1%), and anti-cancer targeted therapy for 21 individuals (37.5%) (**Table 1**). The most common specimen types were BALF (16 cases, 28.6%), followed by blood (13 cases, 23.2%), sputum (13 cases, 23.2%), urine (7 cases, 12.5%), and peritoneal fluid (3 cases, 5.4%) (**Table 1**).

**Table 1:** Demographic and clinical characteristics of 56 cancer patients recruited in this study.

Items	Data
Age (Years, mean ± SD)	61.2 (13.5)
Male (n, %)	37 (66.1%)
Cancer types (n, %)	
Lung cancer	22 (39.3%)
Esophageal cancer	7 (12.5%)
Cervical cancer	5 (8.9%)
Liver cancer	5 (8.9%)
Colorectal cancer	4 (7.1%)
Bladder cancer	2 (3.6%)
Gastric cancer	2 (3.6%)
Lymphoma	2 (3.6%)
Ovarian cancer	2 (3.6%)
Other types^#^	5 (8.9%)
Treatments (n, %)	
Chemotherapy	41 (73.2%)
Radiotherapy	13 (23.2%)
ICIs	18 (32.1%)
Targeted therapy	21 (37.5%)
Sample types (n, %)	
BALF	16 (28.6%)
Blood	13 (23.2%)
Sputum	13 (23.2%)
Urine	7 (12.5%)
Peritoneal fluid	3 (5.4%)
Bile	2 (3.6%)
Pleural fluid	1 (1.8%)
Nasal secretions	1 (1.8%)
Antibiotic use (n, %)	47 (83.9%)
CRP (mg/L, median with IQR)	95.0 (133.9)
White blood cell count (10^9^/L, mean ± SD)	9.5 (7.0)

(^#^Other cancers included acute myeloid leukemia, mediastinal carcinoma, multiple myeloma, nasopharyngeal carcinoma, oral cancer, pancreatic cancer and prostate cancer, and there was one case for each of those cancers. SD, standard deviation; ICIs, immune checkpoint inhibitors; IQR, interquartile range; BALF, bronchoalveolar lavage fluid.)

### Pathogens identified in body fluid samples of immunocompromised cancer patients with suspected infections

Among the 56 body fluid samples collected from immunocompromised cancer patients with suspected infections, 47 (83.9%) samples were identified to have at least one pathogen by nanopore-based metagenomic sequencing or cultures, and no pathogen was detected in the remaining nine (16.1%) specimens (**Supplementary Table 2**). Only 25 (44.6%) samples were identified to have pathogens by culture, suggesting the poor performance of the conventional culture method in detecting pathogens (**Supplementary Table 2**). Remarkably, nanopore-sequencing successfully detected pathogens from 22 out of the 31 culture-negative samples (71.0%) (**Supplementary Table 2**).

All 43 non-blood samples, except one, contained at least one pathogen recognized by either nanopore-based metagenomic sequencing or the culture method (42 cases, 97.7%). Interestingly, among the 42 pathogen-carrying specimens, the culture method failed to find pathogens in approximately 40% of them (17 samples, 40.5%). As for the 13 blood samples, one or more than one type of pathogens was found among five (38.5%) samples by nanopore-based metagenomic sequencing. In contrast, the culture method did not detect pathogens in those samples, suggesting the extremely poor performance of the gold standard method for blood samples.

The pathogens identified by nanopore-sequencing among immunocompromised cancer patients with suspected infections were shown in **Table 2**. Nanopore-sequencing detected bacteria in 42 cases (75.0%), viruses in 10 cases (17.9%), fungi in 16 cases (28.6%), co-infections in 27 cases (48.2%), and fastidious pathogens in eight cases (14.3%) (**Table 2**). The most common pathogen detected was *Escherichia coli* (11 cases, 19.6%), followed by *Stenotrophomonas maltophilia* (9 cases, 16.1%), *Candida albicans* (9 cases, 16.1%), Human gammaherpesvirus 4 (Epstein-Barr virus; 5 cases, 8.9%), *Pseudomonas aeruginosa* (5 cases, 8.9%) and *Haemophilus influenzae* (5 cases, 8.9%), *Klebsiella pneumoniae* (4 cases, 7.1%) and *Pneumocystis jirovecii* (4 cases, 7.1%).

**Table 2:** Summary of pathogens identified by nanopore amplicon sequencing among immunocompromised cancer patients with suspected infections.

Pathogens	All samples (n, %)		Positive in culture (n, %)		Negative in culture (n, %)	
	Total	Positive	Total	Positive	Total	Positive
All pathogens	56	47 (83.9%)	25	25 (100%)	31	22 (71.0%)
Fastidious pathogens^#^	56	8 (14.3%)	0	–^*^	56	8 (14.3%)
Bacteria	56	42 (75.0%)	19	21 (100%)	37	23 (62.2%)
Viruses	56	10 (17.9%)	0	–^*^	56	10 (17.9%)
Fungi	56	16 (28.6%)	8	6 (75.0%)	48	10 (20.8%)
Co-infections	56	27 (48.2%)	7	7 (100%)	49	20 (40.8%)

(^#^Fastidious pathogens included streptococcus pneumoniae, haemophilus influenzae, and moraxella catarrhalis. ^*^Not applicable.)

In the validation experiments, the presence of *Mycobacterium tuberculosis* in the clinical samples from three patients by nanopore-sequencing was all confirmed by commercial RT-PCR diagnostic kits. The presence of fungi (*Candida glabrata*, *Candida albicans*, *Candida tropicalis*, *Pneumocystis jirovecii* and *Aspergillus niger*) in the clinical samples from 16 patients by nanopore-sequencing was also all confirmed by commercial RT-PCR diagnostic kits. Ten DNA viruses included 5 EBV, 4 Human alphaherpesvirus 1 (Herpes simplex virus type 1, HSV1) and 2 HCMV were identified by nanopore-sequencing. The presence of those three DNA viruses including EBV, HSV1 and HCMV had been validated by Sanger sequencing in those clinical samples at the early stage of our study, and Sanger sequencing peak maps were shown in the **Supplementary Figure 2**.

For the false negative findings in culture, 22 out of 31 culture-negative samples were identified to have at least one pathogen by nanopore amplicon sequencing, which proved the high risk of false negative findings in culture. In two samples (No. 17 and No. 33), findings from culture showed the presence of *Candida krusei* and *Proteus mirabilis*. However, those two pathogens were not identified by nanopore amplicon sequencing, which may be caused by the false positive findings in culture or false negative findings in nanopore-sequencing.

### Comparison of the performance of those two pathogen detection methods

**Figure 1:**
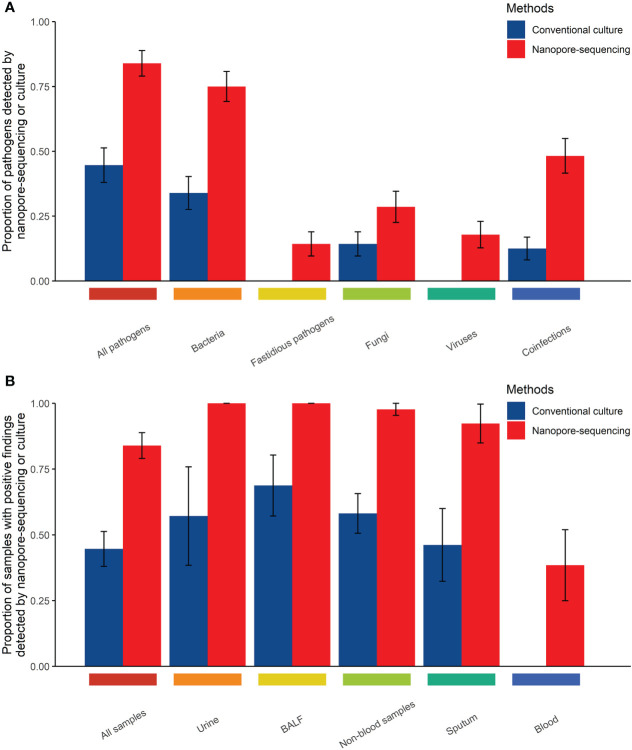
Comparison of the performance of those two methods in detecting pathogens in body fluid samples from immunocompromised cancer patients with suspected infections (**A**, Comparison of the performance of those two methods in detecting pathogens by types of pathogens; **B**, Comparison of the performance of those two methods in detecting pathogens by types of samples).

**Table 3** showed the comparison between nanopore amplicon sequencing and the conventional culture method in their performance of detecting pathogens among immunocompromised cancer patients with suspected infections. Nanopore amplicon sequencing exhibited a significantly high sensitivity than the conventional culture method (83.9% vs. 44.6%, P<0.001), and this advantage existed regardless sample types, blood samples (38.5% vs. 0%, P=0.039) vs. non-blood samples (97.7% vs. 58.1%, P<0.001) (**Table 3**, **Figure 1**). There were 24 cases from which one or more pathogens were detected by nanopore amplicon sequencing; however, no pathogen or an inconsistent finding was found from the cultured specimens (**Supplementary Table 3**).

**Table 3:** Comparison of the performance in detecting pathogens between nanopore amplicon sequencing and conventional culture.

Comparison	Number of patients	Nanopore-sequencing	Conventional culture	P values
All samples	56	47 (83.9%)	25 (44.6%)	<0.001
Subgroup by pathogens				
Fastidious pathogens^*^	56	8 (14.3%)	0 (0%)	0.006
Co-infections	56	27 (48.2%)	7 (12.5%)	<0.001
Bacteria	56	42 (75.0%)	19 (33.9%)	<0.001
Fungi	56	16 (28.6%)	8 (14.3%)	0.065
Viruses	56	10 (17.9%)	0 (0%)	0.001
Subgroup by sample types				
Non-blood samples	43	42 (97.7%)	25 (58.1%)	<0.001
BALF	16	16 (100%)	11 (68.8%)	0.043
Blood	13	5 (38.5%)	0 (0%)	0.039
Sputum	13	12 (92.3%)	6 (46.2%)	0.03
Urine	7	7 (100%)	4 (57.1%)	0.192
Other samples^#^	7	7 (100%)	4 (57.1%)	0.192

*Fastidious pathogens included streptococcus pneumoniae, haemophilus influenzae, and moraxellacatarrhalis. #Other samples included bile, pleural fluid, peritoneal fluid, and nasal secretions.

Compared with the conventional culture method, nanopore amplicon sequencing revealed a significant number of patients with bacterial infections (75.0% vs. 33.9%, P<0.001) or co-infections with other types of pathogens (48.2% vs. 12.5%, P<0.001) (**Table 3**, **Figure 1**). Not surprisingly, nanopore amplicon sequencing detected three common fastidious pathogens (*Streptococcus pneumoniae, Haemophilus influenzae*, and *Moraxella catarrhalis*) from multiple patients, while the conventional culture method failed all cases (14.3% vs. 0.0%, P=0.006). Among 18 fungi-positive samples, nanopore amplicon sequencing successfully identified pathogens in 16 samples, but the conventional culture method only found pathogens from eight patients (88.9% vs 44.4%, P=0.012). Moreover, nanopore amplicon sequencing also detected viruses from 10 samples, which was difficult to be detected *via* the conventional culture method (17.9% vs. 0.0%, P=0.001).

The mean turnaround time (defined as the time from test initiation to the delivery of test results) for this nanopore amplicon sequencing was about 17.5 hours, which was far less than that of the conventional culture method (about 3-5 days).

### Limit of detection

**Figure 2:**
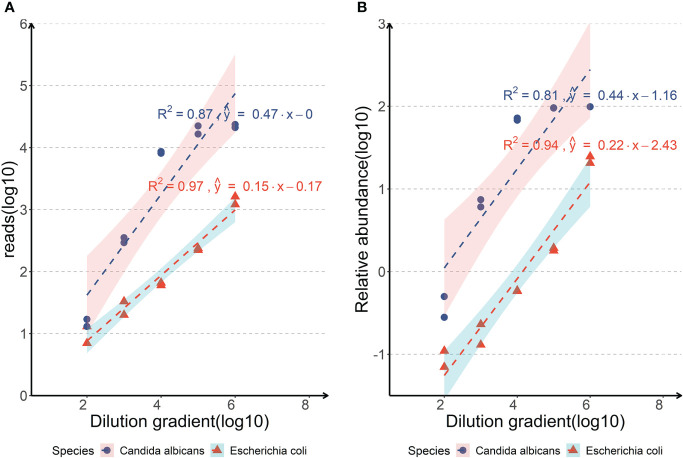
Analyses of the limits of detection of the nanopore amplicon sequencing assay in detecting *Escherichia coli* or *Candida albicans of* body fluid samples. (**A**, Correlations between the reads of the pathogen detected by nanopore amplicon sequencing and the dilution gradient of pathogens spiked in negative sputum; **B**, Correlations between the relative abundance of the pathogen detected by nanopore amplicon sequencing and the dilution gradient of pathogens spiked in negative sputum).

Negative sputum specimens of healthy donors without *Escherichia coli and Candida albicans* in PCR testing and mNGS was used for the LoD test. After trimming the low-quality reads and the reads derived from human DNA, the clean reads were analyzed. The results of sample spiked with *Escherichia coli* or *Candida albicans* indicated that as the amount of input decreased, the reads of the pathogen also decreased (**Figure 2**). The LoD was determined to be 10^3^ copies/ml with two replicates ≥ 10 reads for *Escherichia coli* and 10^2^ copies/ml with two replicates ≥ 10 reads for *Candida albicans*, respectively (**Figure 2**). The LoD of the *Candida albicans* was lower than that of *Escherichia coli* as the detection abundance of fungi was higher than that of bacteria in the same dilution gradient (**Figure 2**).

## Discussion

Rapid and accurate diagnostics of pathogenic microorganisms can enable the early use of appropriate antibiotics and improve patients’ prognosis. Conventional diagnostics of pathogenic microorganisms such as culture have obvious limitations in the management of infections such as long turnaround time and limited sensitivity (Buchan and Ledeboer, 2014; Peri et al., 2022). For RT-qPCR assays or some Sanger sequencing-based assays, a key premise for their successful use is the accurate prediction of suspicious pathogens, which is challenging and requires clinicians to have enough professional qualities (Buchan and Ledeboer, 2014; Church et al., 2020). Moreover, the application of RT-qPCR assays and/or Sanger sequencing-based assays is also limited by throughput and usually cannot cover all suspicious pathogens in clinical practice (Church et al., 2020). Therefore, there is an urgent need for the development of both quick and accurate detection methods of pathogenic microorganisms. In the past decade, metagenomic analyses such as metagenomic next-generation sequencing (mNGS) and nanopore sequencing have emerged as an efficient approach for pathogen detection for patients with infections, which can also detect pathogens in a comprehensive and unbiased way (Chiu and Miller, 2019; Gu et al., 2019). The performance of detecting pathogens under different clinical situations between nanopore sequencing and other methods have also been compared in some clinical studies (Ashikawa et al., 2018; Charalampous et al., 2019; Chan et al., 2020; Deng et al., 2020; Morrison et al., 2020; Fu et al., 2022). Through comparisons with other methods such as culture, RT-qPCR assays and Sanger sequencing-based diagnostics, those studies have confirmed that nanopore sequencing undoubtedly have several advantages such as significantly shorter turnaround time, accurate detection of causative pathogens, simultaneous detection of multiple types of pathogens and accurate detection of antibiotic resistance genes (Ashikawa et al., 2018; Charalampous et al., 2019; Chan et al., 2020; Deng et al., 2020; Morrison et al., 2020; Fu et al., 2022). Therefore, nanopore sequencing has several advantages in detecting causative pathogens compared with RT-qPCR assays or Sanger sequencing-based assays, and can provide more support for the adequate management of infections.

Cancer patients, especially those with immunocompromised status, are at high risk of infections, and there are unmet needs in the early and accurate diagnosis of infections for those patients in clinical practice. This study aimed to evaluate the role of nanopore amplicon sequencing in detecting pathogens among immunocompromised cancer patients with suspected infections. To the best of our knowledge, this is the first study designed to evaluate the role of nanopore amplicon sequencing in pathogen detection among immunocompromised cancer patients. The findings from this study illustrate nanopore amplicon sequencing as a rapid and precise method of pathogen detection. Its superior performance in detecting pathogens may help to improve the outcomes of immunocompromised cancer patients by facilitating the prompt diagnosis of infections and precise anti-infectives treatment.

Both chemotherapy and radiotherapy can significantly increase the risk of infections or infection-related mortality among cancer patients, primarily attributed to the impaired immunity because of the cytotoxic effects of chemotherapy or radiotherapy on hematopoietic stem cells and immune cells (Marchetti and Calandra, 2002; Verma et al., 2016; Wise, 2016; Weil et al., 2018). Prophylactic use of antibiotics before chemotherapy and radiotherapy is a possible infection-control intervention for cancer patients, but its application in the clinic is very limited (Schlesinger et al., 2009; Egan et al., 2019; Van Den Bosch et al., 2021). The adverse effects of antibiotic abuse on human health and the emergence of antibiotic resistance also discourage the prophylactic use of antibiotics as an infection-control intervention for cancer patients (Vento and Cainelli, 2003). Therefore, preventing infections among cancer patients is still a considerable challenge, and effective infection-control strategies are urgently needed.

Early and precise detection of the pathogens is critical to initiating anti-infectives therapy and improving the outcomes of patients with infections, which is difficult to achieve in clinical practice (Bloch and Bailin, 2019; Lamy et al., 2020). Emerging clinical studies in the last five years suggest that sequencing-based metagenomic analyses can detect pathogens earlier and quicker (Rodino et al., 2020; Gu et al., 2021; Piantadosi et al., 2021). Both mNGS and nanopore sequencing have been used to detect pathogens, and have proven to be more efficient than conventional diagnostics of pathogens (Chiu and Miller, 2019). Moreover, metagenomic sequencing-based approaches can also uncover critical information on antibiotic resistance, which is, of course, beneficial for treating patients with infections. Several recent studies further compare the performance of these metagenomic sequencing methods in detecting pathogenic microorganisms under different clinical situations (Schmidt et al., 2017; Votintseva et al., 2017; Gu et al., 2021). Those studies reveal that nanopore sequencing has similar efficiency in detecting pathogens compared with mNGS, but has a shorter turnaround time (Schmidt et al., 2017; Votintseva et al., 2017; Gu et al., 2021). Besides, compared with nanopore sequencing, mNGS have several drawbacks such as longer sequencing time, bulky sequencers and the requirement of sequencing run to be completed before analysis, which impair its application in clinical practice (Rutanga et al., 2018; Chiu and Miller, 2019; Schuele et al., 2021). Therefore, compared with mNGS, nanopore-sequencing can achieve similar sensitivity and accuracy in microbial identification, but it generally requires a less turnaround time, thus making it a more appropriate solution for pathogen detection.

In this study, we evaluated the performance of nanopore amplicon sequencing in parallel with the traditional culture method for detecting pathogens among immunocompromised cancer patients. The results demonstrated that nanopore amplicon sequencing could detect common pathogens such as bacteria, viruses, and fungi. Our sequencing-based assay also uncovered fastidious pathogens and two or more types of pathogens caused co-infections, which generally posed significant challenges for the culture method. Lastly, from receiving samples to generating the test reports, the assay time for nanopore sequencing was approximately 17.5 hours compared to 3-5 days for the culture method, ensuring a rapid detection of pathogens for patients with suspected infections. Therefore, our study shows the feasibility of nanopore amplicon sequencing in rapidly and accurately diagnosing infections among immunocompromised cancer patients.

Improvement in the surveillance of infections may improve supportive care for cancer patients and reduce infection-related mortality. Besides the diagnostic role in suspected infections, metagenomic analyses using next-generation sequencing are also of great interest in infection surveillance. A recent pilot trial by Goggin et al. revealed that some pathogens in cancer patients could be identified by plasma microbial cell-free DNA sequencing (mcfDNA-seq) days before the onset of bloodstream infections, thus enabling early diagnosis and timely treatment (Goggin et al., 2020). However, the performance of nanopore-sequencing in detecting pathogens before the onset of bloodstream infections is still unclear, which should be explored in the future.

In recent years, the spectrum of infections in cancer patients has changed noticeably (El-Sharif et al., 2012; Nesher and Rolston, 2014; Del Castillo et al., 2016). Some studies have shown more invasive fungal infections (Del Castillo et al., 2016; Hardak et al., 2020). Elevated antibiotic resistance among cancer patients with infections has also been reported (Arega et al., 2018). Yet, the current spectrum of infections in cancer patients has not been defined by metagenomic sequencing. We attempt to fill the gap in this study by utilizing nanopore amplicon sequencing (**Table 2**). Our data showed that fungal infections and polymicrobial co-infections were found among 27.1% and 45.8% of cases, respectively. Therefore, the two circumstances should not be ignored when treating cancer patients with suspected infections.

The LoD of the nanopore amplicon sequencing assay was tested on sputum specimen to determine the minimum of bacterial and fungal abundance. We selected sputum specimens as the experimental object mainly because the background microorganisms in sputum specimens were relatively rich, and the content of human-derived host DNA was also considerable, which had a good representative significance. The LoD was determined to be 10^3^ copies/ml with two replicates ≥ 10 reads for *Escherichia coli* and 10^2^ copies/ml with two replicates ≥ 10 reads for *Candida albicans*, respectively (**Figure 2**). Although the LoD of *Escherichia coli* was set up to 10^3^ copies/ml in our experiment, we were able to detect it with a considerable number of reads at 10^2^ copies/ml (7 and 13, respectively). Compared with the detection limits of other nanopore-seq assays from published literatures (arranging from 10^2^ from 10^4^ copies/ml), this nanopore amplicon sequencing assay has at least a non-inferior performance (Imai et al., 2017; Lewandowski et al., 2019; Player et al., 2020; Stefan et al., 2022).

Our study had several limitations. Firstly, the nanopore amplicon sequencing assay had low sensitivity in detecting pathogens in blood samples though it outperformed the conventional culture method. Deep sequencing might improve the sensitivity but indeed with a high-test cost. Secondly, the sample size for immunocompromised cancer patients with suspected infections in this study was not only small but scattered among different types of cancer. Future studies in which more patients of one particular or closely related cancers are recruited need to be performed. Additionally, the sample size was not large enough for specific sample type such as peritoneal fluid. Testing more samples with a better representation is needed to validate the overall performance of nanopore-sequencing. Thirdly, false negative findings in nanopore-sequencing are possible though such possibility is low, and some studies have confirmed the existence of false negative findings in nanopore-sequencing (Charalampous et al., 2019; Gu et al., 2021). Therefore, to reduce the risk of false findings and expand their clinical utility in detecting pathogens, nanopore-sequencing assays still need optimization. Finally, the spectrum of pathogens stratified by sites of infections or types of cancer remains to be determined in future studies.

In conclusion, our study suggests that the nanopore amplicon sequencing assay is a reliable method that allows detecting pathogens among immunocompromised cancer patients with suspected infections rapidly and precisely. Its superior performance in detecting pathogens can help to improve the treatment outcomes for immunocompromised cancer patients. In addition, the utility of nanopore amplicon sequencing in the surveillance or screening of infections among cancer patients should be explored in the future.

## Data availability statement

The datasets presented in this study can be found in online repositories. The names of the repository/repositories and accession number(s) can be found below: Metagenomic sequencing data have been deposited in the NCBI Sequence Read Archive (SRA) (PRJNA854790).

## Ethics statement

The studies involving human participants were reviewed and approved by the ethics committee of the Hefei Cancer Hospital of Chinese Academy of Science and the Cancer Hospital of the University of Chinese Academy of Sciences. The patients/participants provided their written informed consent to participate in this study.

## Author contributions

QD, YC, HG, and HZW designed and supervised the study. QD, YC, XW, AS, and HW enrolled patients and assisted in collection of samples. QD, YW, and HG performed experiments and analyzed data. QD, YC, BW, and HG wrote and edited the manuscript. All authors contributed to the article and approved the submitted version.

## Funding

This study was funded by Research and Development Funding for Medical and Health Institutions (Grant number: 2021YL007) and by the Medical Health Science and Technology Project of Zhejiang Provincial Health Commission (Grant number: 2017188812).

## Conflict of interest

The authors declare that the research was conducted in the absence of any commercial or financial relationships that could be construed as a potential conflict of interest.

## Publisher’s note

All claims expressed in this article are solely those of the authors and do not necessarily represent those of their affiliated organizations, or those of the publisher, the editors and the reviewers. Any product that may be evaluated in this article, or claim that may be made by its manufacturer, is not guaranteed or endorsed by the publisher.

## Supplementary material

Supplementary Table 1 Primers details used for PCR amplification.

Supplementary Table 2 Summary of pathogens identified in body fluid samples of immunocompromised cancer patients with suspected infections (#Other samples included bile, pleural fluid, peritoneal fluid and nasal secretions.)

Supplementary Table 3 Case series of patients with positive findings in nanopore amplicon sequencing but negative or inconsistent findings in cultures. (BALF, bronchoalveolar lavage fluid.)
