# Supplementary material for: Nanopore-based metagenomic sequencing for the rapid and precise detection of pathogens among immunocompromised cancer patients with suspected infections
Source: Front Cell Infect Microbiol. 2022 Sep 20;12:943859. doi: 10.3389/fcimb.2022.943859 (PMC9530710; doi:10.3389/fcimb.2022.943859)
Supplement: Supplementary file 1 [file DataSheet_1.docx]

**
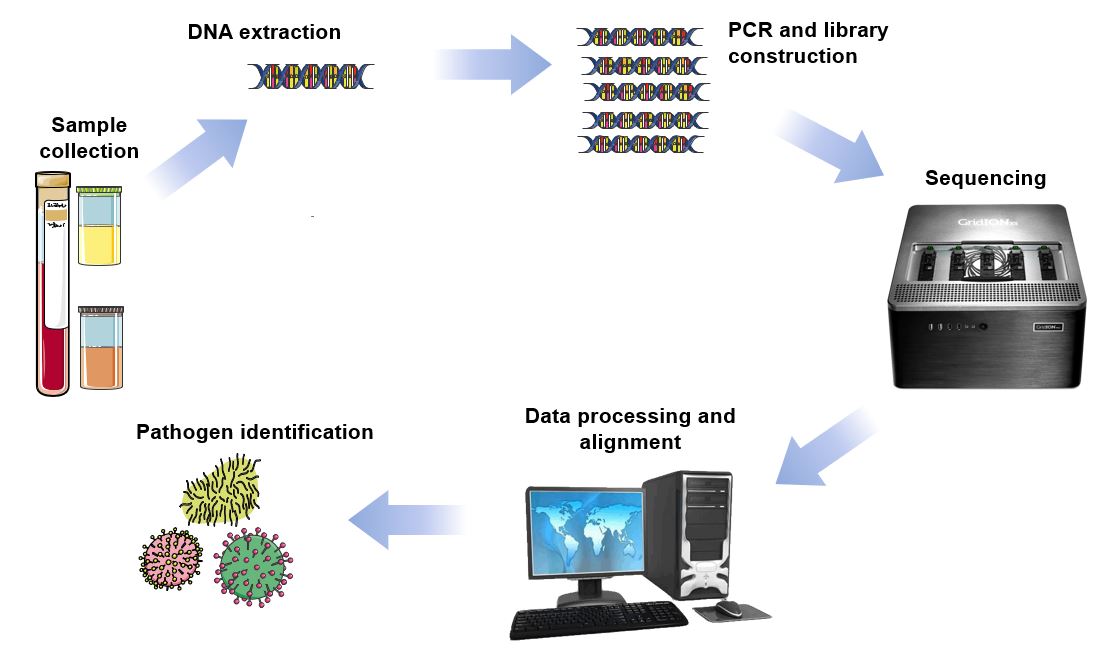
**

**Supplementary figure 1 Schematic workflow of pathogen detection by nanopore amplicon sequencing process**
