# Supplementary material for: Nanopore-based metagenomic sequencing for the rapid and precise detection of pathogens among immunocompromised cancer patients with suspected infections
Source: Front Cell Infect Microbiol. 2022 Sep 20;12:943859. doi: 10.3389/fcimb.2022.943859 (PMC9530710; doi:10.3389/fcimb.2022.943859)
Supplement: Supplementary Table 1 — Primers details used for PCR amplification. [file Table_1.docx]

**Supplementary Table 1 Primers details used for PCR amplification**

| **Items** | **Primer sequence** |
| --- | --- |
| Nano-16S-F27 | TTTCTGTTGGTGCTGATATTGCGRAGAGTTTGATYMTGGCTCAG |
| Nano-16S-R1492 | ACTTGCCTGTCGCTCTATCTTCTACGGYTACCTTGTTAYGACTT |
| Nano-ITS1ngs | TTTCTGTTGGTGCTGATATTGCTCCGTAGGTGAACCTGC |
| Nano-ITS4ngs | ACTTGCCTGTCGCTCTATCTTCCCTSCSCTTANTDATATGC |
| Nano-EBV-Lmp2A-F3 | TTTCTGTTGGTGCTGATATTGCGCTTGGCCCTCTCACTTYTA |
| Nano-EBV-Lmp2A-R3 | ACTTGCCTGTCGCTCTATCTTCCCAGAGGACGAAAGCCAGTA |
| Nano-HCMV-gB-F1 | TTTCTGTTGGTGCTGATATTGCGGCATCATGGTRGTCTACAA |
| Nano-HCMV-gB-R1 | ACTTGCCTGTCGCTCTATCTTCAAATAGCTGGYATTGCGATT |
| Nano-HSV1&2-DNA poly-F2 | TTTCTGTTGGTGCTGATATTGCGATGGCGAGCCACATCTC |
| Nano-HSV1&2-DNA poly-R2 | ACTTGCCTGTCGCTCTATCTTCCGGGCCATGAGCTTGTAATA |
| Nano-HBoV-NP1-F | TTTCTGTTGGTGCTGATATTGCGACGAAGATGAGCTCAGGGA |
| Nano-HBoV-NP1-R | ACTTGCCTGTCGCTCTATCTTCGCTTCTGTCTGTGAGGAAACA |
| Nano-HAdV-HE-F | TTTCTGTTGGTGCTGATATTGCTCYATGCCYAACAGACCCAA |
| Nano-HAdV-HE-R | ACTTGCCTGTCGCTCTATCTTCGCCCGTTCATGTASTCRTAG |
| Nano-VZV-ORF62-F | TTTCTGTTGGTGCTGATATTGCAGGGGGACTGTCTGTGGTC |
| Nano-VZV-ORF62-R | ACTTGCCTGTCGCTCTATCTTCAGCACGTACTGCCGGTATTC |
| Nano-B19-NS1-F | TTTCTGTTGGTGCTGATATTGCCAACCHACCAGGGTAGATCA |
| Nano-B19-NS1-R | ACTTGCCTGTCGCTCTATCTTCCCGACARATGATTCTCCTGAA |
| Nano-HHV6-UL57-F | ACTTGCCTGTCGCTCTATCTTCTGCTCGTTGAKGTTGGAGAT |
| Nano-HHV6-UL57-R | ACTTGCCTGTCGCTCTATCTTCCCTCAGAGCACGAGTATTGT |
| Nano-HHV7-UL10-F | TTTCTGTTGGTGCTGATATTGCGAAAGTCGACAAGAAGTACCCA |
| Nano-HHV7-UL10-R | ACTTGCCTGTCGCTCTATCTTCCTTTTCCATGACAGATCCCGT |
| Nano-HHV8-ORF26-F | TTTCTGTTGGTGCTGATATTGCGGAGATTGCCACCGTTTACA |
| Nano-HHV8-ORF26-R | ACTTGCCTGTCGCTCTATCTTCGTGAGCAGACGGAGACACC |
