# Supplementary material for: Nanopore-based metagenomic sequencing for the rapid and precise detection of pathogens among immunocompromised cancer patients with suspected infections
Source: Front Cell Infect Microbiol. 2022 Sep 20;12:943859. doi: 10.3389/fcimb.2022.943859 (PMC9530710; doi:10.3389/fcimb.2022.943859)
Supplement: Supplementary Table 2 — Summary of pathogens identified in body fluid samples of immunocompromised cancer patients with suspected infections (#Other samples included bile, pleural fluid, peritoneal fluid and nasal secretions.) [file Table_2.docx]

**Supplementary Table 2 Summary of pathogens identified in body fluid samples of immunocompromised cancer patients with suspected infections**

| **Items** | **Number** | **Positive in at least one test** | **Negative in both tests** | **Positive in nanopore-sequencing** | **Positive in culture** | **Positive in both tests** | **Positive in nanopore-sequencing but not in culture** |
| --- | --- | --- | --- | --- | --- | --- | --- |
| Total | 56 | 47 (83.9%) | 9 (16.1%) | 47 (83.9%) | 25 (44.6%) | 25 (44.6%) | 22 (39.3%) |
| Sample types |  |  |  |  |  |  |  |
| Non-blood samples | 43 | 42 (97.7%) | 1 (2.3%) | 42 (97.7%) | 25 (58.1%) | 26 (58.1%) | 17 (39.5%) |
| BALF | 16 | 16 (100%) | 0 (0%) | 16 (100%) | 11 (68.8%) | 11 (68.8%) | 5 (31.2%) |
| Blood | 13 | 5 (38.5%) | 8 (61.5%) | 5 (38.5%) | 0 (0%) | 0 (0%) | 5 (38.5%) |
| Sputum | 13 | 12 (92.3%) | 1 (7.7%) | 12 (92.3%) | 6 (46.2%) | 6 (46.2%) | 6 (50.0%) |
| Urine | 7 | 7 (100%) | 0 (0%) | 7 (100%) | 4 (57.1%) | 4 (57.1%) | 3 (42.9%) |
| Other samples^#^ | 7 | 7 (100%) | 0 (0%) | 7 (100%) | 4 (57.1%) | 4 (57.1%) | 3 (42.9%) |

(Notes: ^#^Other samples included bile, pleural fluid, peritoneal fluid and nasal secretions.)
