# Supplementary material for: Nanopore-based metagenomic sequencing for the rapid and precise detection of pathogens among immunocompromised cancer patients with suspected infections
Source: Front Cell Infect Microbiol. 2022 Sep 20;12:943859. doi: 10.3389/fcimb.2022.943859 (PMC9530710; doi:10.3389/fcimb.2022.943859)
Supplement: Supplementary Table 3 — Case series of patients with positive findings in nanopore amplicon sequencing but negative or inconsistent findings in cultures. (BALF, bronchoalveolar lavage fluid.) [file Table_3.docx]

**Supplementary Table 3 Case series of patients with positive findings in nanopore amplicon sequencing but negative or inconsistent findings in cultures**

| **Case no.** | **Sample type** | **Clinical presentation** | **Nanopore-sequencing result** | **Culture result** |
| --- | --- | --- | --- | --- |
| No.3 | Sputum | Lung cancer; repeated coughing and expectoration | *Haemophilus influenzae* | Negative |
| No.4 | Sputum | Esophageal cancer; repeated coughing and expectoration | *Haemophilus influenzae* | Negative |
| No.17 | Sputum | Esophageal cancer; repeated coughing and expectoration | Human gammaherpesvirus 4 | Candida krusei |
| No.18 | Sputum | Lung cancer; repeated coughing, expectoration and fever | *Haemophilus influenzae* | Negative |
| No.21 | Blood | Colorectal cancer; fever with elevated CRP | *Escherichia coli* | Negative |
| No.24 | Blood | Lung cancer; repeated coughing, expectoration and fever | *Escherichia coli and Haemophilus haemolyticus* | Negative |
| No.27 | Sputum | Lung cancer; coughing and expectoration | *Streptococcus pneumoniae* | Negative |
| No.29 | Blood | Liver cancer; fever with elevated CRP | *Mycobacterium tuberculosis* | Negative |
| No.31 | Sputum | Mediastinal carcinoma; repeated coughing and expectoration | *Pneumocystis jirovecii,* Humanalpha herpesvirus 1 and Humangamma herpesvirus 4 | Negative |
| No.32 | Bile | Gastric cancer; fever with elevated CRP | *Haemophilus influenzae* | Negative |
| No.33 | Urine | Ovarian cancer; lower urinary tract symptom with elevated CRP | *Citrobacter koseri, Escherichia coli* | Proteus mirabilis |
| No.34 | Sputum | Lung cancer; hemoptysis, cough and expectoration | *Moraxella catarrhalis* | Negative |
| No.37 | Urine | Cervical cancer; lower urinary tract symptom with elevated CRP | *Candida albicans* | Negative |
| No.38 | Urine | Cervical cancer; lower urinary tract symptom with elevated CRP | *Streptococcus agalactiae, Candida albicans* | Negative |
| No.48 | Peritoneal fluid | Cervical cancer; acute peritonitis | *Acinetobacter guillouiae* | Negative |
| No.51 | Urine | Esophageal cancer; lower urinary tract symptom with elevated CRP | *Staphylococcus haemolyticus, Fusarium napiforme* | Negative |
| No.53 | Blood | T-cell lymphoma; fever with elevated CRP | *Proteus vulgaris* | Negative |
| No.124 | BALF | Acute myeloid leukemia; severe pneumonia | *Escherichia coli, Candida glabrata and Pneumocystis jirovecii* | Negative |
| No. ZJ04 | Peritoneal fluid | Liver cancer; fever with elevated CRP | *Clostridium perfringens, Bacteroides vulgatus, Escherichia coli, Candida tropicalis, Aspergillus niger* | Negative |
| No. ZJ07 | BALF | Lung cancer; dyspnea with elevated CRP | *Enterococcus faecium, Stenotrophomonas maltophilia, Candida albicans* | Negative |
| No. ZJ08 | BALF | Lung cancer; fever with elevated CRP | *Pneumocystis jirovecii,* Human betaherpesvirus 5 | Negative |
| No. ZJ15 | BALF | Lymphoma; dyspnea with elevated CRP | *Klebsiella pneumoniae, Haemophilus influenzae, Pseudomonas aeruginosa, Enterococcus faecalis, Stenotrophomonas maltophilia,* Human gammaherpesvirus 4 | Negative |
| No. ZJ17 | BALF | Lung cancer; dyspnea with elevated CRP | *Achromobacter xylosoxidans, Stenotrophomonas maltophilia, Candida albicans* | Negative |
| No. ZJ21 | Blood | Ovarian cancer; dyspnea with elevated CRP | *Mycobacterium tuberculosis* | Negative |

(Notes: BALF, bronchoalveolar lavage fluid.)
